# Comments from the Editor of the Special Issue “Clinical Research on Diabetic Complications”

**DOI:** 10.3390/jcm8122193

**Published:** 2019-12-12

**Authors:** Didac Mauricio

**Affiliations:** Department of Endocrinology & Nutrition, CIBER of Diabetes and Associated Metabolic Diseases, Hospital de la Santa Creu i Sant Pau, Autonomous University of Barcelona, 08041 Barcelona, Spain; didacmauricio@gmail.com

**Keywords:** diabetic macroangiopathy, cardiovascular disease, heart disease, cerebrovascular disease, peripheral artery disease, diabetic foot disease, diabetic microangiopathy, diabetic retinopathy, diabetic kidney disease, diabetic neuropathy

## Abstract

With this Editorial, we are hereby presenting to the reader the Special Issue on “Clinical Research on Diabetic Complications”. Chronic complications of diabetes mellitus have a major impact on the life of subjects with the disease, resulting in decreased quality of life and increased morbidity and mortality. This Special Issue includes contributions addressing different clinical aspects of the natural history, prevention and prediction, and characterization and management of diabetes-related complications.

Diabetes mellitus is associated with the development of chronic complications as a result of long-term exposure to hyperglycemia [1]. Diabetes-related complications are roughly divided between those affecting macrovessels (macroangiopathy or macrovascular complications), and those affecting microvessels (microangiopathy or microvascular complications). However, we currently know that we should have a more holistic view, beyond the traditional classification into micro- and macrovascular complications, as diabetes may affect any specific tissue or cell type. We have several articles of the latter concept in this Special Issue (e.g., diabetes-related cognitive impairment). The great burden of diabetes-related complications entails preventing or delaying its appearance as a main goal of diabetes management [2]. 

We have several articles dealing with diabetic macroangiopathy. One of the main pieces of research of this Special Issue, an article by Lin et al., deals with a very relevant topic in the field of cardiovascular disease prevention in diabetes. The authors performed a systematic literature search and a meta-analysis of randomized controlled trials of aspirin use for primary prevention of cardiovascular disease that points to a favorable balance between the risks and benefit of the use of this preventive treatment in individuals with diabetes. In another paper, Castelblanco et al. performed a study using ultrasound tissue characterization of carotid atherosclerotic plaques in which they found that plaques from type 1 diabetic subjects have a different pattern, with increased calcium plaque content, from that of non-diabetic individuals. Further, in another study by the same group, the circulating concentrations of soluble CD36, a potential cardiovascular risk biomarker, was only weakly associated with type 2 diabetes but not with type 1 diabetes. In addition, the work of Li et al. found that the increased arterial stiffness in prediabetes is mainly associated to postprandial glucose (i.e., subjects with impaired glucose tolerance). The proof-of-concept study of Donate-Correa and colleagues showed the potential involvement of the FGF23/Klotho system on the pathogenesis of diabetic foot disease. Finally, regarding diabetes-related cardiovascular disease, in a review paper García-Carro and colleagues wrote an overview of the current strategies and mechanisms of therapies that have demonstrated reno- and cardioprotective effects.

Two original articles contribute to greater insight in the field of diabetic microangiopathic complications. First, Cabré et al. showed that the measurement of dermal electrochemical conductance may be a useful tool to screen diabetic peripheral neuropathy. Further, a paper by Granado-Casas et al. showed that in subjects with type 1 diabetes, those with retinopathy had a poorer quality of life in the absence of other major diabetic complications. 

As pointed out above, diabetes may have a deleterious effect on any cell type. A clear example of this is the impact of diabetes on cognitive function, indicating the metabolic damage on the central nervous system. We have several original articles addressing this issue. In a study by Simó-Servat et al., the authors found gaze fixation abnormalities in subjects with type 2 diabetes (T2D) associated with cognitive status; they concluded that microperimetry to measure parameters of fixation may be a method to detect prodromal stages of dementia. Further, Ogama et al. found that subjects with type 2 diabetes and sarcopenia, among those with cognitive impairment, showed higher glucose variability. Finally, Kim and coworkers provide us with a very interesting piece of work where they describe that the use of dipeptidyl peptidase-IV inhibitors, compared to sulfonylureas, in elderly subjects with type 2 diabetes is associated with a lower risk of dementia in real-world settings.

The rest of the articles of this Special Issue also address relevant questions. A pilot study by Ciudin et al. show the potential of a genetic tool to predict the response to bariatric surgery of subjects with type 2 diabetes. In search of new biomarkers, Del Coco et al. describe a new metabolic serum signature associated with the progression and burden of complications of type 2 diabetes. Finally, Wu et al. use a nationwide database to show that individuals treated with hemodialysis from Taiwan hold a lower risk of developing type 2 diabetes.

To conclude, we should underline that we are far from the optimal knowledge of diabetes-related complications. We need much more research efforts to provide clinicians with new tools to prevent and manage chronic diabetic complications.
